# Phase 1 study of oral selective estrogen receptor degrader (SERD) amcenestrant (SAR439859), in Japanese women with ER-positive and HER2-negative advanced breast cancer (AMEERA-2)

**DOI:** 10.1007/s12282-023-01443-8

**Published:** 2023-03-29

**Authors:** Kenji Tamura, Toru Mukohara, Kan Yonemori, Yumiko Kawabata, Xavier Nicolas, Tomoyuki Tanaka, Hiroji Iwata

**Affiliations:** 1 Shimane University Hospital, Shimane, Japan; 2 National Cancer Center Hospital East, Kashiwa, Japan; 3 National Cancer Center Hospital, Tokyo, Japan; 4 Sanofi, Tokyo, Japan; 5 Sanofi, Montpellier, France; 6 Aichi Cancer Center Hospital, Nagoya, Japan

**Keywords:** Advanced breast cancer, Oral SERD, Amcenestrant, Predictive markers, Pharmacokinetics

## Abstract

**Background:**

This AMEERA-2 study evaluated the pharmacokinetics, efficacy, and safety of the oral selective estrogen receptor degrader amcenestrant as a monotherapy with dose escalation in Japanese postmenopausal women with advanced estrogen receptor-positive and human epidermal growth factor receptor 2-negative breast cancer.

**Methods:**

In this open-label, nonrandomized, phase I study, patients received amcenestrant 400 mg once daily (QD) (*n* = 7) and 300 mg twice daily (BID) (*n* = 3). The incidence of dose-limiting toxicities (DLT), recommended dose, maximum tolerated dose (MTD), pharmacokinetics, efficacy, and safety were assessed.

**Results:**

No DLTs were observed and MTD was not reached in the 400 mg QD group. One DLT (grade 3 maculopapular rash) was reported in a patient treated with 300 mg BID. After repeated oral administration of either dosing regimen, steady state reached before day 8, without accumulation. Four out of 5 response-evaluable patients from 400 mg QD group achieved clinical benefit and showed tumor shrinkage. No clinical benefit was reported in the 300 mg BID group. Overall, most patients (8/10) experienced a treatment-related adverse event (TRAE), with skin and subcutaneous tissue disorders most commonly reported (4/10 patients). No ≥ grade 3 TRAE in 400 mg QD group and 1 grade 3 TRAE in 300 mg BID group were reported.

**Conclusions:**

Amcenestrant 400 mg QD has a favorable safety profile and has been selected as the recommended Phase II dose for monotherapy for evaluating the safety and efficacy of amcenestrant in a larger, global, randomized clinical trial of patients with metastatic breast cancer.

**Trial registration:**

Clinical trial registration NCT03816839.

**Supplementary Information:**

The online version contains supplementary material available at 10.1007/s12282-023-01443-8.

## Introduction

Breast cancer is the most commonly diagnosed cancer in females globally and the fourth highest cause of female cancer-related deaths in Japan [1, 2]. Incidence rates of breast cancer in Japan continue to rise, with an estimated 94,024 cases and 15,700 deaths in 2021 [2]. Treatment strategies for breast cancer subtypes are determined by the tumor expression status of estrogen receptor (ER), progesterone receptor (PgR) and human epidermal growth factor receptor 2 (HER2) [3–5]. Approximately 75–80% of women with breast cancer have ER-positive tumors [6–8] and those with advanced or metastatic ER-positive and HER2-negative (ER + /HER2 −) disease typically receive endocrine therapy [3, 4]. Selective estrogen receptor degraders (SERDs) are an important class of treatment for breast cancer. The SERDs form an unstable SERD-ER complex with reduced mobility which leads to downregulation of ER-‍regulated genes and degradation of the ER protein [9]. Fulvestrant is the only currently approved SERD treatment for locally advanced or metastatic ER-‍positive breast cancer [10, 11]. The FALCON trial has previously demonstrated that fulvestrant can improve progression-free survival compared with oral anastrozole (aromatase inhibitor [AI]) in patients with postmenopausal ER-‍positive advanced or metastatic breast cancer [12]. Fulvestrant has to be administered intramuscularly as it has low permeability and is susceptible to presystemic metabolism, resulting in low bioavailability and suboptimal occupancy of ERs when given orally [13–15]. However, the need for a large injection volume of fulvestrant can cause additional patient burden of injection-related pain and side effects [16]. Such challenges have led to the development of new SERDs with potentially improved oral bioavailability.

Amcenestrant (SAR439859), is a novel, optimized oral SERD with potent dual activity, which antagonizes and degrades the ER resulting in inhibition of the ER signaling pathway [17–19]. Amcenestrant has a fluoropropyl pyrrolidinyl side chain and has demonstrated broad ER antagonist and degrader activities across a large panel of ER-positive tumor cells, including improved inhibition of ER signaling and cell growth. Amcenestrant has also demonstrated significant tumor regression in ER-positive breast cancer in vivo models [17].

Amcenestrant is being assessed in the first in-human, multi-part, Phase I/II AMEERA-1 study in postmenopausal women with pretreated ER + /HER2 − metastatic breast cancer (NCT03284957) [20]. The AMEERA-1 study (Arm 1, Part A; 20–600 mg once daily [QD], N = 16; or 300 mg twice daily [BID], N = 6) investigated amcenestrant dose-escalation and dose expansion (Part B; 400 mg QD, N = 49) as monotherapy and reported no dose-limiting toxicities (DLT) or grade ≥ 3 treatment-related adverse events (TRAE) [20, 21]. In the dose escalation and expansion part of AMEERA-1 (Arm 1; ≥ 150 mg and 400 mg doses, 62 treated patients), the safety profile was deemed to be favorable [22]; all adverse events (AE) were grade 1–2 and hot flush was the most frequent (> 10%) treatment-emergent AE (TEAE). Promising antitumor activity was also reported irrespective of *ESR*1 mutation status.

This AMEERA-2 study aimed to evaluate the safety profile, pharmacokinetics (PK), efficacy, and biomarkers, of amcenestrant, administered orally as a monotherapy with dose escalation, to Japanese postmenopausal women with advanced ER + /HER2 − breast cancer. Additionally, the effects of amcenestrant on ER degradation, through the assessment of tumor biomarker (Ki67, B cell lymphoma 2 [Bcl-2], and PgR) expression, and *ESR1* mutation profiles were investigated.

## Patients and methods

### Study design

AMEERA-2 is an open-label, nonrandomized, Phase I study evaluating amcenestrant monotherapy in Japanese postmenopausal women with ER + /HER2 − advanced breast cancer, conducted at three sites in Japan (NCT03816839). The study protocol was approved by the Institutional Review Board/Institutional Ethics Committees of the participating centers. AMEERA-2 was conducted in accordance with the protocol and the principles expressed in the Declaration of Helsinki, the International Council for harmonisation Guidelines for Good Clinical Practice and Council for International Organizations of Medical Sciences Ethical Guidelines. All patients provided written informed consent prior to the initiation of any study procedures. Two signed informed consent forms were required from each patient, firstly for cycle 1 DLT evaluation and secondly for cycle 2 and subsequent cycles. Protocol deviations were recorded.

### Study population

The study population comprised postmenopausal women aged ≥ 20 years with a histological or cytological proven diagnosis of breast adenocarcinoma, with either evidence of locally advanced disease not amenable to radiation therapy or surgery in a curative intent, or inoperable and/or metastatic disease, with no standardized endocrine treatment option. Patients had previously received ≥ 6 months of endocrine therapy and no more than three chemotherapy regimens for advanced/metastatic disease.

Patients were included in the study either after the primary tumor or metastatic site was confirmed to be (i) ER-positive (> 1% tumor cell staining by immunohistochemistry [IHC] or an Allred score of ≥ 3 by IHC); and (ii) HER2-negative (HER2 non-overexpressing by IHC [0, 1 +] or in situ hybridization-negative [single-probe average HER2 copy number < 4.0 signals/cell] or dual-probe HER2/centromeric probe for chromosome 17 ratio < 2 with an average HER2 copy number < 4.0 signals/cell as per the American Society of Clinical Oncology guidelines [23]).

Any measurable lesions (not mandatory) were assessed in accordance with Response Evaluation Criteria for Solid Tumors version 1.1 (RECIST v1.1). Patients were required to have an Eastern Cooperation Oncology Group performance status (PS) < 2. Patients did not enter the study if they had received curative radiotherapy within 3 weeks before the first administration of amcenestrant. Any patients who had previously received SERDs other than fulvestrant at any time, or fulvestrant within 6 weeks before the first administration of amcenestrant were excluded. Complete inclusion and exclusion criteria are reported in the supplementary material.

In consenting patients, paired tumor samples were collected for molecular analysis from the start of treatment (use of most recent archived biopsy < 3 months of starting treatment or a fresh biopsy was collected at the start of treatment) and the end of cycle 2 (fresh biopsy collected from the primary or metastatic tumor).

### Treatment

Patients received amcenestrant 400 mg QD or 300 mg BID 12 h apart in 28-day cycles until disease progression, unacceptable toxicity, or patient withdrawal, based on the investigator’s decision, or if the patient was lost to follow-up. For the 300 mg BID dosing regimen, amcenestrant was administered once in a fasted state on cycle 1 day 1 (C1D1), then at 12-h intervals (± 2 h) with a 28-day cycle, in fasted or fed state from cycle 1 day 2 onwards and subsequent cycles. For the 400 mg QD dosing regimen, amcenestrant was administered in a fasted state on cycle 1 day 1 (C1D1) and cycle 1 day 22 (C1D22), and in fasted or fed state on Day 2 onwards (except Day 22). Dose escalation from a starting dose of 400 mg QD to 300 mg BID was planned, dependent upon the occurrence of DLT in cycle 1. Each study cohort included three DLT-evaluable patients. For the first cohort, one patient was replaced because of low treatment compliance (< 75%) and was therefore not considered to be DLT evaluable. Thus, while the first cohort included four patients, only three were DLT-evaluable.

Evaluation of dose escalation was undertaken after the last patient in each cohort completed cycle 1. The decision to escalate the dose was made by the study committee based on the dose escalation rule according to the modified toxicity probability interval-2 method and safety profile information from AMEERA-1 [24].

### Outcomes

The primary study objectives were to assess the incidence of DLTs and establish the recommended dose and the maximum tolerated dose (MTD) of amcenestrant monotherapy in Japanese postmenopausal women with ER + /HER2 − advanced breast cancer. Secondary objectives were to characterize the overall safety profile, PK, and antitumor activity of amcenestrant monotherapy. AEs were graded by the National Cancer Institute Common Terminology Criteria for Adverse Events (NCI-CTCAE version 4.03) and classified according to the Medical Dictionary for Regulatory Activities (MedDRA version 23.1). The safety population included all patients who received at least one dose of amcenestrant.

Plasma PK parameters were assessed in the safety population, at both doses, after single (C1D1) or repeated oral administration (C1D22) and reported as descriptive statistics. Additionally, plasma 4β-‍hydroxycholesterol (4β-OH cholesterol) and total cholesterol levels were quantified to assess the potential of amcenestrant to inhibit or induce CYP3A drug metabolizing enzymes.

Antitumoral responses, including confirmed complete response (CR), partial response (PR), and stable disease (SD), or progressive disease were determined by the investigator according to RECIST v1.1. The objective response rate (ORR: CR + PR), clinical benefit rate (CBR: CR + PR + SD ≥ 24 weeks), and non-progression rate at 24 weeks were calculated for each dose level, including 90% confidence intervals (CI). Response duration (time from initial response to the first documented tumor progression) was calculated for each patient. The ‘response-evaluable’ population included patients who had measurable lesions at baseline and ≥ 1 evaluable tumor assessment.

### Biomarker analysis

Exploratory objectives included gene mutational profiling of tumors over time in cell-free DNA (cfDNA) and the analysis of biomarker expression in tumor tissues. Twelve independent mutations of the *ESR1* gene, including hotspot mutations, were identified in all patients using a multiplex droplet digital polymerase chain reaction assay for plasma-extracted cfDNA samples at study baseline and the end of cycle 2. Mutation profiles in cfDNA samples for all patients were identified at baseline and end-of-treatment (EOT). ER levels were determined by IHC performed centrally on patients’ tumor samples. ER degradation was assessed by comparing the change between baseline and at end of cycle 2 ER levels. Changes in expression of breast cancer biomarkers (Ki67, Bcl-2, and PgR) were also assessed by IHC performed at baseline and at the end of cycle 2.

## Results

### Patients and treatment

**Fig. 1:**
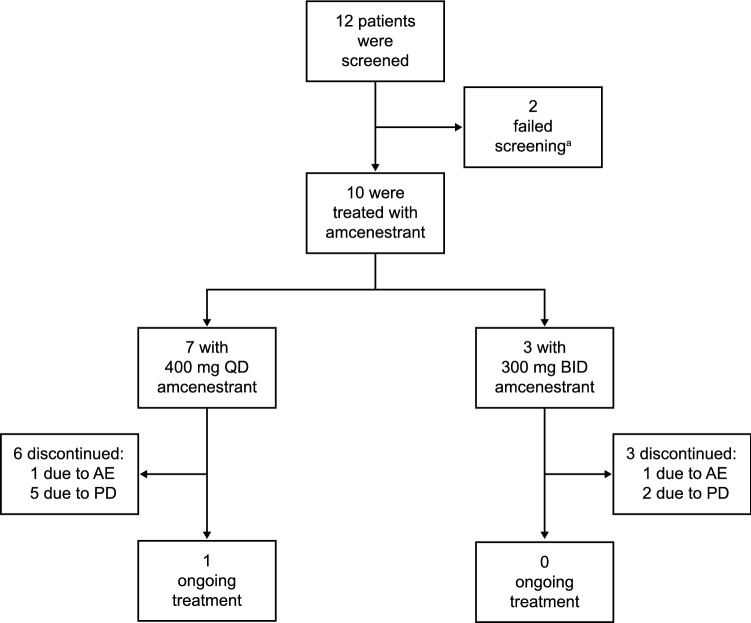
Patient disposition. *AE* adverse event, *BID* twice daily, *PD* progressive disease, *QD* once daily. ^a^One patient failed screening due to inadequate renal function and one patient failed postmenopausal criteria

The AMEERA-2 study began on 25 March 2019 and ran until the data cut-off date of 30 March 2021. A total of 12 patients were screened and 10 were treated with amcenestrant (Fig. 1). Seven patients were treated with amcenestrant 400 mg QD and three were treated with amcenestrant 300 mg BID. One patient did not have a measurable lesion at baseline. At data cut-off, one patient remained on treatment with amcenestrant 400 mg QD. The main reason cited for treatment discontinuation was disease progression (7 of 10 patients: 5 from the 400 mg QD group and 2 from the 300 mg BID group; 70%). Two patients, one from each treatment group, discontinued due to serious AEs.

Median patient age was 67.0 years (range 48–76 years) and nine (90%) patients had metastatic breast cancer (Table 1). The main organ involved in metastatic breast cancer was bone, reported for seven (70%) patients. All patients had received ≥ 2 (range 2–9) prior treatments for advanced-stage cancer. All patients were pretreated with ≥ 2 prior lines of endocrine therapy and had previously received AI therapy in the advanced setting. Seven (70%) patients had received prior targeted therapy (6 received CDK 4/6 inhibitors), and five (50%) had received prior chemotherapy. Six (60%) patients had each previously received SERDs and selective estrogen receptor modulators.

**Table 1:** Patient demographics and baseline characteristics (safety population)

Characteristic	All patients (*n* = 10)	Amcenestrant 400 mg QD (*n* = 7)	Amcenestrant 300 mg BID (*n* = 3)
Age, years			
Median (range)	67.0 (48–76)	72.0 (54–76)	65.0 (48–67)
≥ 65	7 (70.0)	5 (71.4)	2 (66.7)
ECOG PS			
0	7 (70.0)	5 (71.4)	2 (66.7)
1	3 (30.0)	2 (28.6)	1 (33.3)
Median time (range) from first diagnosis to amcenestrant treatment, years	8.4 (3.4–22.0)	8.7 (3.4–18.4)	7.9 (3.9–22.0)
Histology			
Adenocarcinoma	5 (50.0)	4 (57.1)	1 (33.3)
Other	5 (50.0)	3 (42.9)	2 (66.7)
Invasive ductal carcinoma	4 (40.0)	3 (42.9)	1 (33.3)
Scirrhous carcinoma	1 (10.0)	0	1 (33.3)
Disease stage at diagnosis			
I	1 (10.0)	1 (14.3)	0
II	6 (60.0)	3 (42.9)	3 (100)
III	1 (10.0)	1 (14.3)	0
IV	2 (20.0)	2 (28.6)	0
Extent of disease			
Primary	1 (10.0)	1 (14.3)	0
Locally advanced	0	0	0
Metastatic	9 (90.0)	6 (85.7)	3 (100)
Median number of organs with metastases	3 (1–4)	3 (1–4)	3 (3–3)
Organs with metastases			
Bone	7 (70.0)	4 (57.1)	3 (100)
Breast	3 (30.0)	3 (42.9)	0
Colon	1 (10.0)	1 (14.3)	0
Liver	5 (50.0)	3 (42.9)	2 (66.7)
Lung	4 (40.0)	2 (28.6)	2 (66.7)
Lymph node	5 (50.0)	4 (57.1)	1 (33.3)
Skin	2 (20.0)	2 (28.6)	0
Other	2 (20.0)	1 (14.3)	1 (33.3)
Intent of prior therapy			
Advanced only	2 (20.0)	2 (28.6)	0
Adjuvant/advanced	7 (70.0)	5 (71.4)	2 (66.7)
Number of prior lines of therapy in advanced setting			
Range	2–9	2–6	3–9
≤ 1	0	0	0
2	4 (40.0)	4 (57.1)	0
3	2 (20.0)	1 (14.3)	1 (33.3)
> 3	4 (40.0)	2 (28.6)	2 (66.7)
Endocrine resistance status			
Primary resistance^a^	1 (10.0)	0	1 (33.3)
Secondary resistance^b^	9 (90.0)	7 (100)	2 (66.7)
Prior anticancer therapy in advanced settings			
Chemotherapy	5 (50.0)	2 (28.6)	3 (100)
Hormonotherapy	10 (100)	7 (100)	3 (100)
Immunotherapy	0	0	0
Targeted therapy	7 (70.0)	5 (71.4)	2 (66.7)
Other	1 (10.0)	1 (14.3)	0
Types of hormone therapy in the advanced setting			
SERM	6 (60.0)	4 (57.1)	2 (66.7)
SERD	6 (60.0)	4 (57.1)	2 (66.7)
AI	10 (100)	7 (100)	3 (100)
Other	4 (40.0)	2 (28.6)	2 (66.7)

Data are *n* (%) unless otherwise stated. Type of prior anticancer treatment according to customized drug grouping from World Health Organization Drug Dictionary Enhanced (WHO-DDE) 2020 September 1. Data for disease location, histology type, and stage were collected at initial diagnosis

*AI* aromatase inhibitors, *BID* twice daily, *ECOG PS* eastern cooperation oncology group performance status, *QD* once daily, *SERD* selective estrogen receptor degrader, *SERM* selective estrogen receptor modulator

^a^Relapse < 24 months after the start of adjuvant hormonotherapy, for patients without advanced hormonotherapy treatment; progression < 6 months after the start of the last prior advanced hormonotherapy, for patients with advanced hormonotherapy treatment

^b^Relapse ≥ 24 months after the start and < 12 months after the end of adjuvant hormonotherapy, for patients without advanced hormonotherapy treatment; progression ≥ 6 months after the start of the last prior advanced hormonotherapy, for patients with advanced hormonotherapy treatment

### Dosing and safety

The median (range) duration of amcenestrant, across both doses was 16.1 weeks (1–78 weeks); 25.3 weeks (3–78 weeks) for the 400 mg QD group and 4.6 weeks (1–7 weeks) for the 300 mg BID group. The median (range) relative dose intensity across both doses was 97.1% (74–100%); 99.5% (74–100%) for the 400 mg QD and 88.2% (87–100%) for 300 mg BID group. Five (71.4%) patients in the 400 mg QD group and one (33.3%) patient in the 300 mg BID group had at least one dose omission.

No DLTs were observed and MTD was not reached in the amcenestrant 400 mg QD group. Overall, most patients (8 of 10 [80%] patients) experienced a TRAE (Table 2), with skin and subcutaneous tissue disorders most commonly reported (4 of 10 [40%] patients). No trend for any specific AEs was observed. While no grade ≥ 3 TEAEs or TRAEs were reported in the 400 mg QD group, three (30%) patients experienced any-grade TEAEs leading to either dose reductions or omissions: these were single events of a gastric cancer (not treatment-related), palmar-plantar erythrocythemia syndrome (grade 2, treatment-related) and rash (grade 2, treatment-related). One DLT, a grade 3 maculopapular rash deemed to be a serious TRAE, was observed in the 300 mg BID group leading to permanent treatment discontinuation for that patient after 1 week of treatment. One patient had a grade ≥ 3 TEAE of gamma-glutamyl-transferase increase (not treatment-related) in the 300 mg BID group. No treatment-related cardiac toxicity and no bradycardia or QTc prolongation were observed. No deaths were reported during the treatment period.

**Table 2:** Treatment-related adverse events

SOC/Preferred Term	All patients (*n* = 10)		Amcenestrant 400 mg QD (*n* = 7)		Amcenestrant 300 mg BID (*n* = 3)	
	All grades	Grade ≥ 3	All grades	Grade ≥ 3	All grades	Grade ≥ 3
Any	8 (80.0)	1 (10.0)	6 (85.7)	0	2 (66.7)	1 (33.3)
Nervous system disorders	2 (20.0)	0	1 (14.3)	0	1 (33.3	0
Dysgeusia	1 (10.0)	0	1 (14.3)	0	0	0
Headache	1 (10.0)	0	0	0	1 (33.3)	0
Gastrointestinal disorders	2 (20.0)	0	2 (28.6)	0	0	0
Constipation	1 (10.0)	0	1 (14.3)	0	0	0
Nausea	1 (10.0)	0	1 (14.3)	0	0	0
Skin and subcutaneous tissue disorders	4 (40.0)	1 (10.0)	3 (42.9)	0	1 (33.3)	1 (33.3)
Dermatitis acneiform	1 (10.0)	0	1 (14.3)	0	0	0
PPES	1 (10.0)	0	1 (14.3)	0	0	0
Rash	1 (10.0)	0	1 (14.3)	0	0	0
Maculopapular rash	1 (10.0)	1 (10.0)	0	0	1 (33.3)	1 (33.3)
Musculoskeletal and connective tissue disorders	1 (10.0)	0	1 (14.3)	0	0	0
Arthritis	1 (10.0)	0	1 (14.3)	0	0	0
General disorders and administration site conditions	2 (20.0)	0	1 (14.3)	0	1 (33.3)	0
Fatigue	1 (10.0)	0	1 (14.3)	0	0	0
Malaise	1 (10.0)	0	0	0	1 (33.3)	0
Injury, poisoning and procedural complications	1 (10.0)	0	1(14.3)	0	0	0
Sunburn	1 (10.0)	0	1 (14.3)	0	0	0

Data are *n* (%) of patients with amcenestrant related TEAEs (worst grade) listed by MedDRA (version 23.1) SOC and preferred terms and graded by NCI CTCAE version 4.03. Data are listed by SOC internationally agreed order and preferred term and sorted by decreasing frequency in ‘all grades’ in ‘all patients’

*BID* twice daily*, MedDRA* medical dictionary for regulatory activities*, NCI CTCAE* national cancer institute common terminology criteria for adverse events, *PPES* palmar-plantar erythrodysaesthesia syndrome*, QD* once daily*, SOC* system organ class

### PK variables

During the PK data assessment there were no issues with administration that may have impaired the measurements. All patients from safety population were evaluable for PK parameters, except one patient at 400 mg QD on C1D22 due to dose omission, and one patient at 300 mg BID on C1D22 due to early termination. Amcenestrant was absorbed without any T_lag_ and after repeated doses of 400 mg QD or 300 mg BID, T_max_ was similar to that after the respective single dose administration ranging between 2.90 and 4.43 h (Table 3). CLss/F was low for 400 mg QD and 300 mg BID dosing, regardless of food intake, and no accumulation was observed after repeated dosing. Mean ± standard deviation (SD) C_trough_ levels were similar over cycle 1 for 400 mg QD repeated administration, from day 2 (556 ± 572 ng/mL) to day 22 (348 ± 83.7 ng/mL), with no accumulation (Supplementary Fig S1). For amcenestrant 300 mg BID, mean ± SD C_trough_ levels were 574 ± 241 ng/mL at day 2 and 1260 ± 63.6 ng/mL at day 22, reaching a maximum by day 8 (Supplementary Fig S1). No significant schedule effects were observed on systemic exposure at steady-state when comparing 400 mg QD and 300 mg BID, which corresponded to a 1.5-fold increase in daily dose intensity, apart from C_trough_ levels. The 300 mg BID regimen resulted in a C_max_ value 27% lower (point estimate [PE], 0.730; 90% CI 0.415–1.284), AUC_0-24 h_ 14% higher (PE, 1.138; 90% CI 0.747–1.732), and C_trough_ 269% higher (PE, 3.692; 90% CI 2.628–5.187), compared with the 400 mg QD dose.

**Table 3:** Amcenestrant plasma pharmacokinetic parameters after single and repeated doses

Parameter (unit)	Amcenestrant 400 mg QD		Amcenestrant 300 mg BID	
	Day 1	Day 22	Day 1	Day 22
T_max_ (h)				
*n*	7	6^a^	3	2^b^
Median (min–max)	2.90 (1.95–4.05)	2.97 (1.92–5.97)	4.00 (4.00–5.57)	4.43 (2.85–6.00)
C_max_ (ng/mL)				
*n*	7	6	3	2
Mean ± SD	5800 ± 1840	5020 ± 1290	5130 ± 2180	3940 ± 2380
G_mean_ (CV)	5500 (31.7)	4880 (25.7)	4820 (42.5)	3560 (60.3)
AUC_tau_ (h*ng/mL)^c^				
*n*	7	6	3^d^	2
Mean ± SD	51,300 ± 20,800	40,100 ± 5650	35,700 ± 15,800	24,500 ± 13,300
G_mean_ (CV)	48,500 (40.5)	39,800 (14.1)	33,300 (44.4)	22,600 (54.2)
CLss/F (L/h)				
*n*	NA	6	NA	2
Mean ± SD	NA	10.1 ± 1.37	NA	14.4 ± 7.78
G_mean_ (CV)	NA	10.1 (13.5)	NA	13.3 (54.2)

*AUC*_*tau*_ area under the plasma concentration, *BID* twice daily, *CLss/F* apparent total body clearance after repeated extra-vascular doses at steady state from the plasma, *C*_*max*_ maximum concentration observed, *G*_*mean*_ geometric mean, *NA* not applicable, *QD* once daily, *T*_*max*_ time to maximum plasma concentration

^a^One patient not included

^b^One patient discontinued after day 9

^c^Dosing interval (TAU) = 24 h

^d^Patients in the 300 mg BID treatment arm received 300 mg QD for cycle 1 day1

The 4β-OH cholesterol ratio measured after 4 weeks of multiple amcenestrant doses suggested potential for induction at a 400 mg QD dose, and a higher effect at 300 mg BID dose. Compared with baseline levels, 4β-‍OH cholesterol concentrations were increased from 3 weeks following amcenestrant dosing. Geometric means of C1D22/C1D1 ratio of 4β-OH cholesterol were 1.6 (90% CI 1.4–2.0) and 2.9 (90% CI 0.246–33.7) for 400 mg QD and 300 mg BID doses, respectively, and the cycle 2 day 1 /C1D1 ratios were 2.3 (90% CI 1.844–2.877) and 3.4 (90% CI 0.280–40.61) for 400 mg QD and 300 mg BID, respectively. The 4β-‍OH cholesterol/total cholesterol showed a similar ratio, indicating no bias was introduced via any possible direct effect on total cholesterol by amcenestrant.

### Antitumor activity

**Fig. 2:**
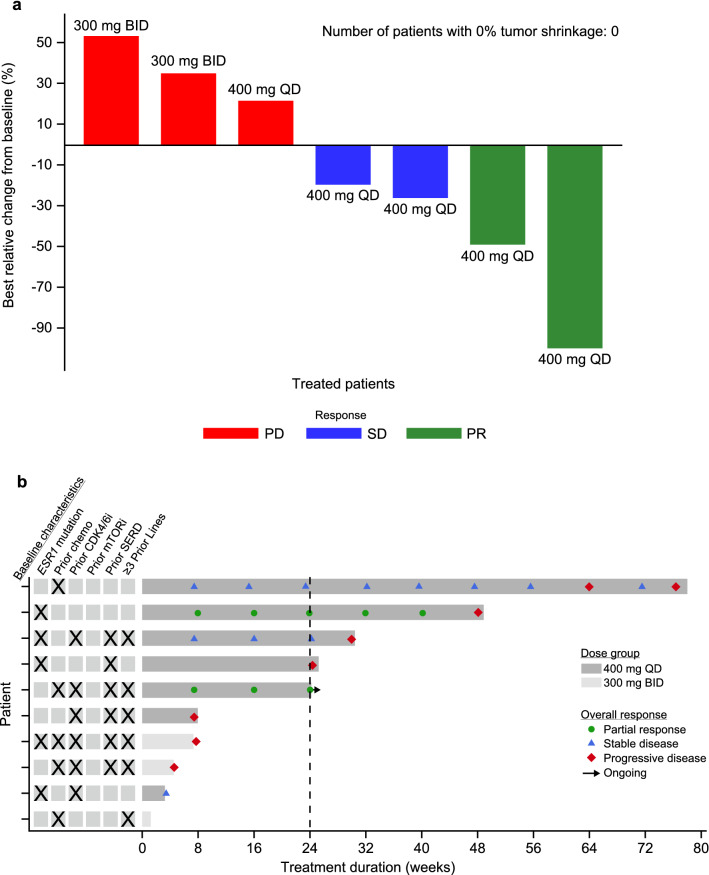
Waterfall and swimmer plots. **a** Waterfall plot of best relative change from baseline in the sum of diameters of target lesions in the response-evaluable population by local investigators/radiologists review (*n* = 7; two patients were missing relative change/confirmation data and one patient had no target lesion) and **b** Swimmer plot of duration of treatment in the safety population (*n* = 10) with overall responses assessed by local investigators/radiologists review. *PD* progressive disease, *SD* stable disease, *PR* partial response, *chemo* chemotherapy, *CDK4/6i* cyclin-dependent kinase 4/6 inhibitor, *mTORi* mammalian target of rapamycin inhibitor, *SERD* selective estrogen receptor degrader. Checkboxes correspond to baseline characteristics

A total of seven patients were response-evaluable: five from the 400 mg QD group and two from the 300 mg BID group. In the 400 mg QD group, two of five patients achieved a clinical response (ORR: 40.0%) and four of five patients achieved a clinical benefit (CBR: 80.0%). No clinical response (ORR and CRB) was reported in the 300 mg BID group. In the 400 mg QD group, two (40.0%) patients had confirmed PR, one of these patients achieved a duration of response of 40.4 weeks and the other patient achieved it for 16.9 weeks and remains in the study, with the duration of response continuing to be assessed. One of these 2 patients was pre‍-‍treated with fulvestrant. Two (40%) patients from the 400 mg QD group had SD, of which, one patient was pre-treated with fulvestrant. In the 300 mg BID group, both (100%) patients had progressive disease (Fig. 2).

In the response-evaluable population, tumor shrinkage (relative change in tumor size from baseline to best overall response) was observed in four of five (80%) patients receiving amcenestrant 400 mg QD (and four of seven [57.1%] patients with a response to either dose), with one patient showing > 90% shrinkage of her target lesion (Fig. 2).

### Biomarker analysis

**Fig. 3:**
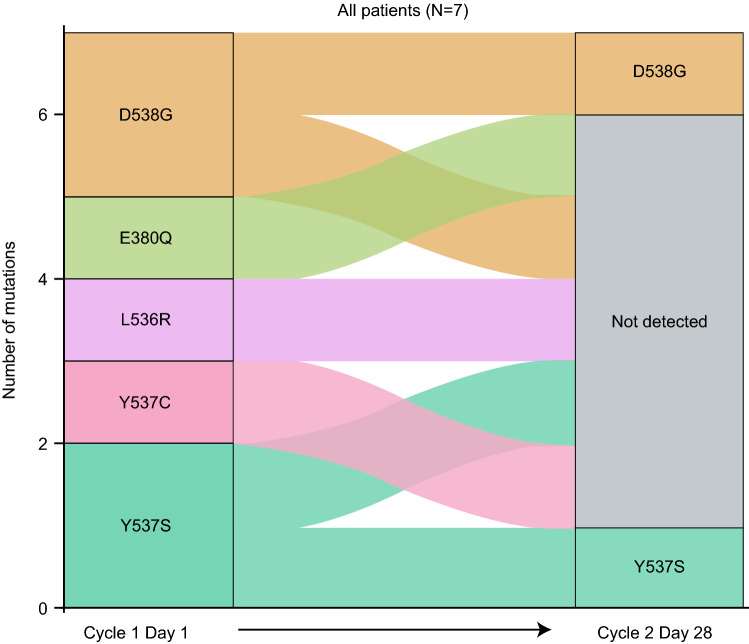
Evolution of all patients with *ESR1* mutations over time (safety population with available data at baseline and cycle 2 day 28)

At baseline, from the seven safety-evaluable patients with available data at baseline and cycle 2 day 28 (C2D28), four patients had *ESR1* mutations in cfDNA, including treatment-resistant *D538G* and *Y537S* mutations, and three had *ESR1* wild type. At C2D28, amcenestrant had reduced the number of most of the *ESR1* mutations; detectable in two patients and not detectable in five patients. Patients with *ESR1* mutations treated with amcenestrant demonstrated decreased allele frequency at C2D28 for at least one mutation. From *ESR1* mutations detected at baseline, 71% were not detectable at C2D28 (Fig. 3). In addition, two of three patients with *ESR1* mutations at baseline achieved clinical benefit among pooled response-evaluable patients (*n* = 6) with available data at baseline and C2D28, including patients who had resilient *D538G* and *Y537S* mutations. Of note, the patient with *ESR1* mutations who failed to achieve any clinical benefit had a higher allele frequency than the other patients at baseline.

Amcenestrant demonstrated robust trends of antitumor activity as shown by overall reductions (expressed as median relative change from screening) in ER protein expression (− 46.4%; range, − 100% to 1733%; *n* = 5), reduction in PgR expression (− 99.2%; range, − 100% to − 78.7%; *n* = 3), reduction in Ki67 expression (− 25%; range, − 90% to 33.3%; *n* = 4), and reduction in ER activation score by gene set variation analysis (− 0.4; range, − 0.8 to 0.6; *n* = 5).

There was an overall increase in Bcl-2 expression (H-score, IHC; median relative change from screening: 56.2%; range, − 55.6% to 275%; *n* = 4) at C2D28, although no specific trend was observed in changes of cytoplasmic Bcl-2 H-score in relation to clinical benefit (data not shown).

## Discussion

The AMEERA-2 study has demonstrated that amcenestrant has a favorable safety profile in Japanese postmenopausal women with ER + /HER2 − advanced breast cancer, with no grade ≥ 3 TEAEs at a dose of 400 mg QD and a PK profile similar to the one previously observed in a global population of patients (AMEERA-1, Arm 1, Part A). Amcenestrant 400 mg QD has subsequently been selected as the recommended Phase II dose for monotherapy. The previous AMEERA-1 dose escalation study (Arm 1, Part A) which assessed amcenestrant doses of 20, 150, 200, 400, and 600 mg QD showed that amcenestrant was rapidly absorbed (median T_max_ of approximately 3 h) [20, 21]. AMEERA-1 also demonstrated that following repeated amcenestrant administration up to 600 mg QD, the PK profile showed little or no accumulation. Notably, the amcenestrant 400 mg QD dose resulted in median ER occupancy of 100% and achieved the mean C_trough_ value across the dose range [21]. Based on these data, it was hypothesized that amcenestrant 300 mg BID would have a higher probability of ER saturation than a 600 mg QD dose. The amcenestrant 400 mg QD dose was selected for expansion, supported by a lack of DLTs, favorable safety profile, and the MTD not being previously reached [20–22, 25].

In AMEERA-2, most patients were aged > 65 years, had metastases, and were heavily pre-treated (prior therapy received: hormonal therapy and/or chemotherapy and/or targeted therapy). While no DLT was reported in patients treated with amcenestrant 400 mg QD, one DLT (grade 3 maculopapular rash) was reported in a patient treated with amcenestrant 300 mg BID. These findings contrast with those from the AMEERA-1 study wherein no DLTs and no related grade ≥ 3 events were reported with amcenestrant up to 600 mg QD and 300 mg BID [21, 22].

The preliminary safety profile of amcenestrant in Japanese postmenopausal women in AMEERA-2 was generally comparable with that reported for the AMEERA-1 study [20–22], and for fulvestrant in other studies including those in Japanese patients [26, 27]. In AMEERA-2, the most common TRAEs were skin and subcutaneous tissue disorders reported in four (40%) patients with dermatitis acneiform, palmar-plantar erythrodysaesthesia syndrome, rash, and maculopapular rash occurring in one patient each. Two patients discontinued study treatment due to TRAEs, one patient from the 300 mg BID group (grade 3 maculopapular rash) and one from the 400 mg QD group (grade 2 rash). In the 400 mg QD group, all TRAEs were either grade 1 or 2. No clinically significant, cardiac TRAEs were observed. While a higher proportion of treatment-related skin and subcutaneous tissue events were reported in the AMEERA-2 study than in AMEERA-1, the low patient and event numbers warrant consideration.

In the present study, amcenestrant absorption was observed without T_lag_, while a median T_max_ ranging from 3–4 h was recorded following repeated doses of both regimens. After repeated oral administration of either dosing regimen, steady state reached before day 8, without accumulation. Mean apparent oral clearance of amcenestrant was low at steady state and consistent across doses. C_max_ and AUC_0-24 h_ were similar between the administered doses, with a higher C_trough_ for the 300 mg BID regimen. PK data were similar to those reported in patients from the AMEERA-1 study [20–22]. The 4β-OH cholesterol ratios reported in AMEERA-2 suggest potential for an induction of CYP3A activity by amcenestrant at both dose levels.

Antitumor activity was demonstrated in seven evaluable patients (ORR: two [28.6%] out of seven patients; CBR: four [57.1%] out of seven patients), and this was comparable with that reported in AMEERA-1 part B (ORR and CBR: five [10.9%] and thirteen [28.3%] out of 46 patients respectively) [21]. The antitumor effects of amcenestrant were demonstrated by the ER degradation and inhibition of ER signaling, and decrease in *ESR1* mutated alleles post treatment. These observations align with the AMEERA-1 study observations [21, 28]. Four (57.1%) of the seven patients in AMEERA‍-‍2 had *ESR1* mutations detected in cfDNA, two of whom had baseline *D538G* and *Y537S* mutations located in the ligand binding domain of the ER protein. These specific mutations may be of clinical relevance as they are associated with resistance to endocrine therapy in vitro and may also influence tumor sensitivity to endocrine therapy in patients [7]. These findings are in concordance with the response to amcenestrant in patients harboring *D538G* and *Y537S* mutations from the AMEERA-1 study and in preclinical studies of amcenestrant [17, 21].

Small number of patients is a limitation of AMERA-2 study and therefore, caution is required when making any conclusions based on the available antitumor activity and biomarker data, with all findings requiring further evaluation and confirmation.

In summary, data from AMEERA-2 provide a basis for evaluating the safety and efficacy of 400 mg QD amcenestrant in a larger, global, randomized clinical trial of patients with metastatic breast cancer, including those from Japan (AMEERA-3; NCT04059484). However, AMEERA-3 trial did not meet primary endpoint of improving progression-free survival. Further, in AMEERA-5 trial, amcenestrant did not meet the prespecified boundary for continuation and therefore, Sanofi has discontinued the global clinical development program of amcenestrant.

## Supplementary Information

Below is the link to the electronic supplementary material.

Supplementary file1 (DOCX 50 KB)

## Data Availability

Qualified researchers may request access to patient level data and related study documents including the clinical study report, study protocol with any amendments, blank case report form, statistical analysis plan, and dataset specifications. Patient level data will be anonymized and study documents will be redacted to protect the privacy of our trial participants. Further details on Sanofi’s data sharing criteria, eligible studies, and process for requesting access can be found at: https://www.vivli.org/.
