# Supplementary material for: Phase 1 study of oral selective estrogen receptor degrader (SERD) amcenestrant (SAR439859), in Japanese women with ER-positive and HER2-negative advanced breast cancer (AMEERA-2)
Source: Breast Cancer. 2023 Mar 29;30(3):506–17. doi: 10.1007/s12282-023-01443-8 (PMC10119216; doi:10.1007/s12282-023-01443-8)
Supplement: Supplementary file 1 — Supplementary file1 (DOCX 50 KB) [file 12282_2023_1443_MOESM1_ESM.docx]

**Phase 1 study of oral selective estrogen receptor degrader (SERD) amcenestrant (SAR439859), in Japanese women with ER-positive and HER2-negative advanced breast cancer (AMEERA-2)**

Kenji Tamura^1^, Toru Mukohara^2^, Kan Yonemori^3^, Yumiko Kawabata^4^, Xavier Nicolas^5^, Tomoyuki Tanaka^4^, Hiroji Iwata^6^

^1^ Shimane University Hospital, Shimane, Japan

^2^ National Cancer Center Hospital East, Kashiwa, Japan

^3^ National Cancer Center Hospital, Tokyo, Japan

^4^ Sanofi, Tokyo, Japan

^5^ Sanofi, Montpellier, France

^6^ Aichi Cancer Center Hospital, Nagoya, Japan

**Corresponding author:** Dr. Hiroji Iwata

Address: Aichi Cancer Center Hospital, Nagoya, Japan

Telephone: 090-052-762-6111

Email: [hiwata@aichi-cc.jp](mailto:hiwata@aichi-cc.jp)

**Supplementary figure S1** Amcenestrant plasma C_trough_ concentration profile


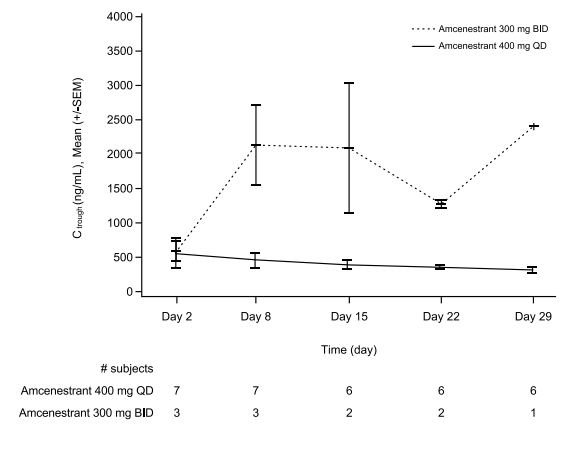


Mean (SEM) amcenestrant plasma C_trough_ concentration-time-profiles after repeated administration of 300 mg BID or 400 mg QD doses. *BID* twice daily, *QD* once daily, *SEM* standard error of mean

**Patients and Methods**

Inclusion criteria

Patients were eligible for inclusion in the study only if all of the following criteria applied:

*Age*

- Aged ≥ 20 years old, at the time of signing the informed consent.

*Type of participant and disease characteristics*

- Patients with histological or cytological proven diagnosis of adenocarcinoma of the breast with evidence of either locally advanced not amenable to radiation therapy or surgery in a curative intent, inoperable and/or metastatic disease and with no standardized endocrine treatment option by investigators’ judgement.
- Either the primary tumor or any metastatic site was positive for estrogen receptor (ER) (> 1% tumor cell staining by immunohistochemistry [IHC] or an Allred score of ≥ 3 by IHC consistent with local standards).
- Either the primary tumor or any metastatic site was HER2 non-overexpressing by IHC (0, 1+) or in situ hybridization-negative based on single-probe average HER2 copy number <4.0 signals/cell or dual-probe HER2/centromeric probe for chromosome 17 (CEP17) ratio < 2 with an average HER2 copy number <4.0 signals/cell as per the American Society of Clinical Oncology guidelines.
- Prior chemotherapy for advanced/metastatic disease was allowed: participants must have received no more than 3 prior chemotherapeutic regimens.
- Patients must have received at least 6 months of endocrine therapy for advanced breast cancer in the past. Last prior anticancer therapy (either hormonal or chemotherapy or targeted therapy) could have been discontinued for any reason.
- Measurable lesion preferable by Response Evaluation Criteria for Solid Tumors (RECIST) 1.1.
- For patients who consented to paired biopsies (before treatment and during treatment): for baseline samples, formalin-fixed and paraffin-embedded (FFPE) archived biopsy sample could be used, but preferably fresh biopsies from primary or recurrence or metastasis were to be collected. It was recommended that the end of cycle 2 biopsy was collected at the same location as the baseline biopsy, whenever possible and tumor accessible for a biopsy during treatment.

*Sex*

Female postmenopausal women as defined by one of the following:

- With spontaneous cessation of menses > 12 months prior to registration in the absence of chemotherapy, tamoxifen and toremifene.
- Or with cessation of menses of duration ≤ 12 months or secondary to hysterectomy and had follicle stimulating hormone (FSH) level in the postmenopausal range according to institutional standards (or >34.4 IU/L if institutional range was not available) prior to registration.
- Or who had received hormonal replacement therapy but had discontinued this treatment and had FSH level in the postmenopausal range according to institutional standards (or > 34.4 IU/L if institutional range is not available) prior to registration.
- Or with status post bilateral surgical oophorectomy.
- Or were premenopausal women on a gonadotropin-releasing hormone (GnRH) analog for at least 6 months (to be continued during study treatment) and had a negative pregnancy test prior to initiation of study treatment and at monthly intervals during treatment.

*Informed Consent*

- Were capable of giving signed informed consent including compliance with the requirements and restrictions listed in the informed consent form (ICF) and in the study protocol.

Exclusion criteria

Patients who met all the above inclusion criteria were screened for the following exclusion criteria. Patients were excluded from the study if any of the following criteria applied:

*Medical conditions*

- Eastern Cooperative Oncology Group (ECOG) performance status (PS) ≥2
- Significant concomitant illness, including psychiatric condition that, in the opinion of the Investigator or Sponsor, would have adversely affected the patient’s participation in the study.
- Medical history or ongoing gastrointestinal disorders potentially affecting the absorption of amcenestrant. Patients who were unable to swallow normally and to take capsules. Predictable poor compliance to oral treatment.
- Any malignancy related to human immunodeficiency virus (HIV), or unresolved viral hepatitis.
- Patients with a life expectancy less than 3 months.
- Participants with any other cancer. However, adequately treated basal cell or squamous cell skin cancer or in situ cervical cancer or any other cancer from which the participant had been disease free for > 3 years were allowed.
- Patients with known brain metastases, leptomeningeal carcinomatosis or/and spinal cord compression. Those with brain metastases that had been previously totally resected or irradiated were eligible provided no progression or relapse was observed within 4 weeks of the treatment.
- Patients with known endometrial disorders, including evidence of endometrial hyperplasia, dysfunctional uterine bleeding or ovarian cysts.
- Patients with Gilbert disease.
- Non-resolution of any prior treatment-related toxicity to < grade 2, except for alopecia according to National Cancer Institute Common Terminology Criteria for adverse events (NCI-CTCAE) v4.03.

*Prior/concomitant therapy*

- Major surgery within 4 weeks prior to first study treatment administration.
- Treatment with atazanavir, lopinavir (antiviral agents) ketoconazole (antifungal), quercetin (antioxidant), dabigatran (anticoagulant), digoxin (cardiac glycoside), and fexofenadine (antihistamine) less than 2 weeks before first study treatment administration or 5 elimination half-lives whichever was longest.
- Treatment with strong and moderate CYP3A inhibitors/inducers within 2 weeks before first study treatment administration or 5 elimination half-lives whichever is longest.
- Treatment with anticancer agents (including investigational drugs) less than 2 weeks before first study treatment administration (less than 4 weeks if the anticancer agents were antibodies).
- Prior treatment with another selective ER down-regulator (SERD) except fulvestrant for which a washout of at least 6 weeks was required prior to the first study drug administration.
- Treatment with curative radiotherapy less than 3 weeks before first study treatment administration.

*Diagnostic assessments*

- Inadequate hematological function including neutrophils < 1.5 x 10^9^/L; platelet count < 100 x 10^9^/L (use of G-CSF and/or platelet transfusion was not allowed within 1 week prior to the screening test. Use of Pegylated G-CSF is not allowed within 2 weeks prior to the screening test.
- Prothrombin time: International normalized ratio (INR) > 1.5 times the upper limit of normal (ULN). For patients who received anticoagulant therapy, INR should have been within therapeutic range.
- Inadequate renal function with serum creatinine ≥ 1.5 x ULN or between 1.0 and 1.5 x ULN with estimated glomerular filtration rate (eGFR) < 60 mL/min/1.73 m2 as estimated using the abbreviated Modification of Diet in Renal Disease formula.
- Liver function: aspartate aminotransferase (AST) > 3 x ULN, or alanine aminotransferase (ALT) >3 x ULN. Alkaline phosphatase (ALP) up to grade 2 (2.5 to 5xULN) was acceptable only if related to the presence of bone and/or liver metastases as judged by the Investigator. Total bilirubin > 1.5 x ULN.

*Other exclusions*

- Patient not suitable for participation, whatever the reason, as judged by the Investigator, including medical or clinical conditions, or participants potentially at risk of noncompliance to the study procedures (i.e., unwillingness and inability to comply with scheduled visits, drug administration plan, laboratory tests, other study procedures, and study restriction).
- Patient was the Investigator or any sub investigator, research assistant, pharmacist, study coordinator, or other staff or relative thereof directly involved in the conduct of the protocol.
- Individuals accommodated in an institution because of regulatory or legal order; prisoners or subjects who were legally institutionalized.
- Patients were dependent on the Sponsor or Investigator (in conjunction with section 1.61 of the ICH Good Clinical Practice (GCP) Ordinance E6).
- Participants who were employees of the clinical study site or other individuals directly involved in the conduct of the study, or immediate family members of such individuals.
- Any specific situation during study implementation/course that may rise ethics considerations.
- Sensitivity to any of the study interventions, or components thereof, or drug or other allergy that, in the opinion of the Investigator, contraindicated participation in the study.
